# A Comparison of the Antioxidant Effects Between Hydrogen Gas Inhalation and Vitamin C Supplementation in Response to a 60-Min Treadmill Exercise in Rat Gastrocnemius Muscle

**DOI:** 10.3389/fphys.2021.745194

**Published:** 2021-10-14

**Authors:** Li Chaoqun, Zhao Yuqi, Zhou Shi, Yu Zhenghui, Wen Li

**Affiliations:** ^1^School of Sports and Health, Nanjing Sport Institute, Nanjing, China; ^2^School of Kinesiology, Shanghai University of Sport, Shanghai, China; ^3^Institute of Exercise and Health, Tianjin University of Sport, Tianjin, China; ^4^Discipline of Sport and Exercise Science, Faculty of Health, Southern Cross University, Lismore, NSW, Australia

**Keywords:** hydrogen inhalation, reactive oxygen species, oxidative stress, antioxidants, mitochondrial biogenesis, aerobic exercise

## Abstract

The reactive oxygen species (ROS) produced during exercise act as a double-edged sword because they may cause oxidative damage but also play a role in the signaling pathways. A supplementation of exogenous antioxidants can reduce the total amount of ROS during exercise while it may also affect the ROS’ role in the signaling pathways of mitochondrial biogenesis. It has been suggested that hydrogen gas, as an antioxidant, can selectively scavenge hydroxyl radicals but does not affect superoxide anion’s signal transduction. The aim of this study was to compare the effects of 1-h hydrogen gas inhalation 30min prior to a treadmill exercise on the key biomarkers of mitochondrial biogenesis and related signaling pathways, and the activities of endogenous antioxidant enzymes, with those of vitamin C, in the rat skeletal muscle. Eighty-one 8-week-old male Sprague–Dawley (SD) rats were randomly assigned to three interventions (exercise-only, exercise+4%H_2_, and exercise+vitamin C at 500mg/kg weight, with 27 rats under each intervention), and sampled at pre-, immediately post and 4h post a 60-min treadmill exercise at speed of 27m/min and flat inclination, with nine rats in each sub-group. Expression of mitochondrial biogenetic markers and related signaling molecules in gastrocnemius muscle, and concentrations of oxidative stress markers in serum were measured. Two-way ANOVA or Kruskal–Wallis analyses showed that both hydrogen inhalation and vitamin C supplementation significantly reduced serum levels of MDA immediately after exercise and AGEs 4h after exercise. The pre-exercise supplement of vitamin C significantly reduced mitochondrial complex IV concentrations and PGC-1α, NRF-1, TFAM gene expression levels compared to the pre-exercise group, but the hydrogen gas intervention did not result in a reduction in these measurements. Unlike vitamin C, hydrogen inhalation did not blunt post-exercise mitochondrial biogenetic signals, but resulted in an increase in complex IV concentration, activation of PGC-1α, and TFAM and NRF-2 gene transcription, and up-regulation of PGC-1α protein expression. The findings indicated that hydrogen gas inhalation could play the role as an effective antioxidant in response to the exercise, whilst it did not significantly affect mitochondrial biogenesis. The dose–response relationship and antioxidant effects in different types of exercise for hydrogen inhalation require further investigation.

## Introduction

There has been evidence that high intensity exercise, either prolonged or short-duration, can cause an increase in reactive oxygen species (ROS) level in skeletal muscle (Powers et al., 2016). According to the free radical theory of aging, an increased formation of ROS may cause cumulative damage to the cell and hence the organism (Harman, 1956). This view has been widely acknowledged, and supported by the evidence that high concentrations of ROS play a role in insulin resistance, mitochondrial dysfunction, diabetes mellitus, obesity, inflammation and infection (Alfadda and Sallam, 2012). Consistently with Harman’s theory (1956), evidence has emerged in recent years that an unaccustomed and/or exhaustive exercise can generate excessive ROS, leading to oxidative stress-related tissue damage and impaired muscle contractility (He et al., 2016). One of the counter measures is the supplementation with antioxidants, such as vitamin C, vitamin E, or alpha lipoic acid, etc. (Margaritelis et al., 2018). However, conflicting evidence has been shown in the literature (Merry and Ristow, 2016a; de Oliveira et al., 2019; Ismaeel et al., 2019). A meta-analysis of 11 studies, including humans and rodent models and various exercise modalities, reported that supplementation with vitamins C and E for three or more weeks produced inconsistent results, with six studies showing no effect and two studies reporting negative effects on mitochondrial metabolism and exercise performance (Nikolaidis et al., 2012). This means exogenous antioxidant supplementation may blunt exercise-induced beneficial adaptations (Strobel et al., 2011; Paulsen et al., 2014; Ristow, 2014). ROS also serve as signaling molecules to regulate physiological processes and partly mediate health benefits of exercise (Schieber and Chandel, 2014). An increased ROS level within the mitochondria can result in an adaptive response that subsequently enhances resistance to oxidative stress, and may cause a long-term reduction of oxidative stress (Ristow and Zarse, 2010). Therefore, further research is required to explore the pros and cons of various antioxidant supplementation regimes for optimizing adaptations to exercise and benefits to health.

Mitochondrial biogenesis is a process by which new mitochondria are produced from existing mitochondria. The peroxisome proliferator-activated receptor-gamma (PPARγ) coactivator-1alpha (PGC-1α) is known as a core regulator of the mitochondrial biogenesis (Scarpulla, 2011). Once PGC-1 is activated by either phosphorylation or de-acetylation, it activates nuclear respiratory factors 1 and 2 (NRF-1 and NRF-2), and subsequently mitochondrial transcription factor A (TFAM). The activation of this PGC-1α/NRF-1/TFAM pathway leads to the synthesis of mitochondrial DNA and proteins and generation of new mitochondria (Li et al., 2017). Interestingly, these signal molecules are all related to the redox state in the cell. ROS, especially hydrogen peroxide (H_2_O_2_), have been confirmed to act as signaling molecules, regulating the transcription of genes and affecting protein modification in *ex vivo* experiments (D'Autréaux and Toledano, 2007; Sies, 2017). Previous studies have found that the addition of H_2_O_2_ to lung fibroblasts can cause an increase in the expression of NRF-1 (Lee et al., 2002). The lipopolysaccharide-induced oxidative damage also leads to an upregulation of NRF-1 and NRF-2, that activates mitochondrial biogenesis and expression of antioxidant enzymes, among other physiological processes (Suliman et al., 2003). Application of H_2_O_2_ to mouse embryonic cells can increase the levels of PGC-1α and PGC-1β mRNA, which may be necessary for motion-induced mitochondrial biogenesis (St-Pierre et al., 2006). In addition, some factors in the upstream signaling pathway of PGC-1 are also redox-sensitive proteins, such as AMP-activated protein kinase (AMPK) and cyclic AMP (cAMP)-response element-binding protein (CREB; Thirupathi and de Souza, 2017; Bouchez and Devin, 2019). Together, these lines of evidence suggest a potential regulatory network between ROS and mitochondrial biogenesis.

Supplementation of antioxidants directly reduces the total amount of ROS in skeletal muscle, including H_2_O_2_ as a signaling molecule, which could blunt the exercise-induced mitochondrial biogenesis signal. For example, oral supplementation of vitamin C can inhibit exercise-induced PGC-1α expression and mitochondrial biogenesis in skeletal muscle (Gomez-Cabrera et al., 2008). In addition, vitamin C supplementation can also inhibit exercise-induced expression of several antioxidant enzymes in human muscle such as catalase (CAT) and superoxide dismutase (SOD; Gomez-Cabrera et al., 2008). Whether supplementation of antioxidants during exercise is beneficial has been debated due to the reported negative effects, such as vitamin C on exercise adaptation (Peternelj and Coombes, 2011). Hydrogen gas inhalation has been proposed as an antioxidant intervention for reducing acute exercise-induced inflammation and oxidative stress (Nogueira et al., 2018; Kawamura et al., 2020). However, available evidence on the antioxidant effect of H_2_ inhalation is still limited to date. Ohsawa et al. (2007) reported that inhalation of H_2_ as an antioxidant did not reduce ^•^O_2_^−^, but selectively scavenged the strongly oxidizing ^•^OH and a small amount of peroxynitrite that causes most of the oxidative damage in cells (Nogueira and Branco, 2021). Meanwhile, H_2_ inhalation does not appear to have known side effects while it can be more easily absorbed as a small molecule (Nogueira and Branco, 2021).

Therefore, considering the above-mentioned controversial effects of vitamin C supplementation and the possible benefits of H_2_ inhalation, the aim of this preliminary study was to compare the potential antioxidant effect of H_2_ inhalation 30min prior to a bout of treadmill exercise with that of vitamin C supplementation, using a rodent experimental model. Because different types of ROS may play different roles in regulating adaptations to exercise and responses to injuries (Li et al., 2016; Sies, 2017), it was hypothesized that hydrogen inhalation as an antioxidant intervention can effectively attenuate the markers of oxidative stress induced by the exercise while having little interference with the cellular signaling role of the ROS. The outcomes of the study may provide new evidence for the potential implications of this intervention, and shed light for further investigations.

## Materials and Methods

### Experimental Design

Male Sprague–Dawley (SD) rats were randomly and equally assigned into three types of interventions with 27 rats in each type: exercise-only (as control, CE), vitamin C supplement and exercise (VCE), and hydrogen inhalation and exercise (HE). Rats under each type of intervention were randomly divided into three sub-groups that were sampled during resting as the baseline control (CE0, VCE0, HE0), immediately post a treadmill exercise (CE1, VCE1, and HE1) and 4h post the exercise (CE4, VCE4, and HE4), with nine animals in each sub-group and a total number of 81.

G-power 3.1 software was used to calculate the sample size. When set for the effect size of 0.35, *α* level of 0.05, and power of 0.80, 82 subjects would be required for a two-way ANOVA analysis. The total number of animals used was 81 for a consistant number in each of the nine subgroups, which was determined also with reference to a study, in which a similar statistical approach was employed (Wadley and McConell, 2010), and a consideration of the laboratory capacity.

Prior to the exercise, rats were given either hydrogen or vitamin C, or no supplementation. The exercise was performed on a motor-driven treadmill (Beijing ZhiShouDuoBao Biological Technology Co. Ltd.) for 60min. At the end of the exercise, the rats were anesthetized by intraperitoneal injection of pentobarbital sodium (50mg/kg). Blood sample was collected by cardiac puncture, and serum was prepared for analysis of the selected variables. The gastrocnemius muscle tissues were excised and immediately snap frozen in liquid nitrogen before subsequent analysis.

### Ethics Approval Statement

The work was performed in strict accordance with the *Guideline on Administration of Lab Animals* and *Guidelines on the Humane Treatment of Laboratory Animals* issued by the Ministry of Science and Technology of China. The study was approved by the Animal Care and Use Committee of Tianjin University of Sport, China (approval number TJUS2019023).

### Animals

The SD rats were obtained from the Vital River Laboratory Animal Technology Co., Ltd. (Beijing, China). All rats were 8-week-old males and weighed 279.3±20.6 grams when purchased. The animals were housed in pairs in clean cages with free access to commercial rodent food (Vital River, China) and water. The room was kept at a temperature of 21±1°C, humidity of 55±10% and light 12h on and 12h off. All animals were acclimatized to the environment for 1week.

### Treadmill Exercise

The rats were given three familiarization sessions for running on a motor-driven treadmill on separate days within 1week, with running speed of 15m/min on flat surface for 15min. The formal experiment was performed 3days after the last familiarization session. The treadmill speed was set at 27m/min and slope at zero degree. This workload was approximately equivalent to 75% of maximum oxygen consumption that would be within the range for aerobic endurance training for SD rats (Bedford et al., 1979). The rats were required to run continuously for 60min. Electrical stimulation was used to encourage rats complying with the protocol.

### Vitamin C Supplementation

The animals in the VCE groups received vitamin C solution (Solaibao, Beijing, China) at a dosage of 500mg/kg body weight, which is equivalent to 0.06mg/cm^2^ body surface area (BSA) with reference to a previous study (Gomez-Cabrera et al., 2008). The vitamin C solution was delivered to the VCE group by gavage 1h before exercise, while the CE and HE groups received an equal volume of physiological saline (The HE group was gavage before hydrogen absorption).

### Hydrogen Gas Inhalation

The animals in the HE groups were placed in a ventilation chamber for 1h where the air containing 4% hydrogen gas was continuously supplied. It has been shown in the literature that hydrogen concentration in skeletal muscles reaches the peak level after 1h of inhalation (Liu et al., 2015). The hydrogen gas was produced by an X9 Hydrogen Nebulizer (Five Cloud, Beijing). The oxygen concentration in the chamber was maintained in a range (20–23%) similar to that in the normal air. The rats were removed from the chamber 30min before the commencement of the treadmill exercise, and they breathed normal air during the exercise.

### Variables Measured

#### Gene Transcription

Total RNA was extracted from the gastrocnemius muscle sample using Trizol (Invitrogen, United States)/chloroform procedure. cDNA solutions were prepared using the Revertaid First Strand cDNA Biogenesis Kit (Thermo Scientific, United States). The primers were designed by General Biological Co., Ltd. (China), verified in primer blast at the National Center for Biotechnology Information. For each pair of primers, we performed melt curve analysis and agarose electrophoresis of the amplicons to ensure that a single product of the calculated size was amplified. RT-PCR was finally performed in 7500 Real-Time Fluorescent Quantitative PCR System (Applied Biosystems, United States) using the ChamQ Universal SYBR qPCR Master Mix (Vazyme, Nanjing, China). The fractional cycle number at which fluorescence passed the threshold (CT values) was used for quantification using the comparative CT method. Sample values were normalized to the threshold value of a housekeeping gene GAPDH: ΔCT (experiment)=CT (experiment)−CT (GAPDH). The CT value of the CE0 was used as the reference. ΔΔCT=ΔCT (experiment)−ΔCT (CE0). mRNA expression was calculated by the following equation: fold change=2^−ΔΔCT^. Primer sequences are shown in Table 1.

**Table 1:** Primer sequences.

Genes	Forward (5'→3')	Reverse (5'→3')
NRF-1	GTCGCTCATCCAGGTTGGTA	CTCTGATGCTTGCGTCGTCT
NRF-2	CCCCATTGTCCAATTCTTCA	CTGCGGTAACCACTTCCTCT
PGC-1	ACCCACAGGATCAGAACAAACC	GACAAATGCTCTTTGCTTTATTGC
TFAM	AGCCATGTGGAGGGAGCTT	TTGTACACCTTCCACTCAGCTTTAA
Mn-SOD	TGGACAAACCTGAGCCCTAA	GACCCAAAGTCACGCTTGATA
CAT	AAGCTGGTTAATGCGAATGG	TTGAAAAGATCTCGGAGGCC
GPx	CGACATCGAACCCGATATAGA	ATGCCTTAGGGGTTGCTAGG
COX1	GGAACCCTCTACCTATTATTTGG	GTGGTACAAGTCAGTTCCCGAA
COX4	CCAGGGATGAGAAAGTCCAAT-3'	CACCCAGTCACGATCAAAGG

#### Protein Expression

To analyze the expression of PGC-1α, TFAM, AMPKα1, cAMP, CREB and p53 protein, frozen gastrocnemius muscle samples were homogenized with cell lysate and protease inhibitors added. After ultrasonic fragmentation standing at 4°C for 30min, followed by centrifugation at 12,000*g* for 15min, the supernatant was extracted. The concentration of the extracted protein solution was determined using BCA Protein Assay Kit (Solarbio, Beijing, China). The protein solution was adjusted to the same concentration and then heated to deform, and store at −20°C after cooling. The proteins of interest were separated by electrophoresis using SDS-PAGEs protein gels, blocking with nonfat milk. According to the molecular weight of the target proteins, GAPDH or β-tublin was selected as internal reference to avoid their overlap. The bands were incubated with antibodies to make the proteins fluoresce. Bands were film developed in a dark room and relative intensities were quantified using ImageJ v1.8.0 after film scanning, and normalized to GAPDH or β-tublin intensity obtained from the same blot after stripping.

#### Enzyme-Linked Immunosorbent Assay

The cytochrome c oxidase (complex IV) in the muscle and advanced glycation end products (AGEs) concentrations in the serum samples were calculated by measuring the absorbance at a wavelength of 450nm, using rat cytochrome c oxidase ELISA kit and rat advanced glycation end products, AGE ELISA kit (CUSABIO, Wuhan, China), according to the instructions in the kits.

#### SOD Activity

The SOD viability of samples was determined using SOD typing assay kit (Jiancheng, Nanjing, China) following the kit’s instructions. The total SOD (T-SOD) was measured according to the inhibition of sample SOD on the production of nitrite by xanthine and xanthine oxidase reaction system, followed by the determination of CuZn-SOD activity after Mn-SOD activity treatment. The Mn-SOD activity was obtained by the subtraction of the two values (T-SOD – CuZn-SOD).

#### Mitochondrial Aconitase Activity

ACO catalyzed conversion of citrate to isocitrate was detected using ACO activity assay kit (Comin, Suzhou, China). The oxidative decarboxylation of isocitrate reduced NAD^+^ to NADH, resulting in the rise of light absorption at 340nm, which reflected the ACO activity of the sample.

#### Serum Malondialdehyde

A malondialdehyde content kit (Comin, Suzhou, China) was used according to the condensation of MDA with thiobarbituric acid (TBA), to generate a red product, with a maximum absorption peak at 532nm. Colorimetric measurement was performed to estimate the content of peroxidized lipids in the samples.

### Statistical Analysis

A two-way ANOVA was performed for the effect of intervention (control, vitamin C, H_2_) and exercise (pre, immediately post, and 4h post) and their interaction. Since the animals were sampled at different time points, the nine subgroups were treated as independent groups. The Shapiro–Wilk test was performed on all data sets for normal distribution. Square root transformation was performed for data sets that failed the normality test to satisfy the assumptions of ANOVA, or Kruskal–Wallis test was performed for the data sets that still failed the normality test after transformation. When a significant main effect or an interaction was found, *post hoc* analysis with Bonferroni adjustment was used for pairwise comparisons, with the alpha level set at 0.05 level. All statistical tests were conducted using SPSS (ver.19.0), and figures were created by GraphPad Prism (ver.9.0.2). Mean value and SD of the variables were reported unless otherwise stated.

## Results

Mitochondrial cytochrome C oxidase (complex IV) concentration and expression of mitochondrial biogenetic signal-related proteins and genes (PGC-1α, TFAM, NRF-1, etc.) in gastrocnemius muscle of each group were measured to reflect the level of mitochondrial biogenesis. The activity and gene expression of antioxidant enzymes (SOD, CAT, and GPx) in the gastrocnemius muscle were assessed to reflect the effects of vitamin C and hydrogen on the antioxidant system. The biomarkers of lipid peroxidation and protein peroxidation in the serum (MDA and AGEs) were examined to reflect the ability of the two antioxidants in reducing oxidative stress after acute exercise. Due to technical errors, some variables had missing values as indicated in the captions of the figures or tables.

### Mitochondrial Complex IV Synthesis

**Figure 1:**
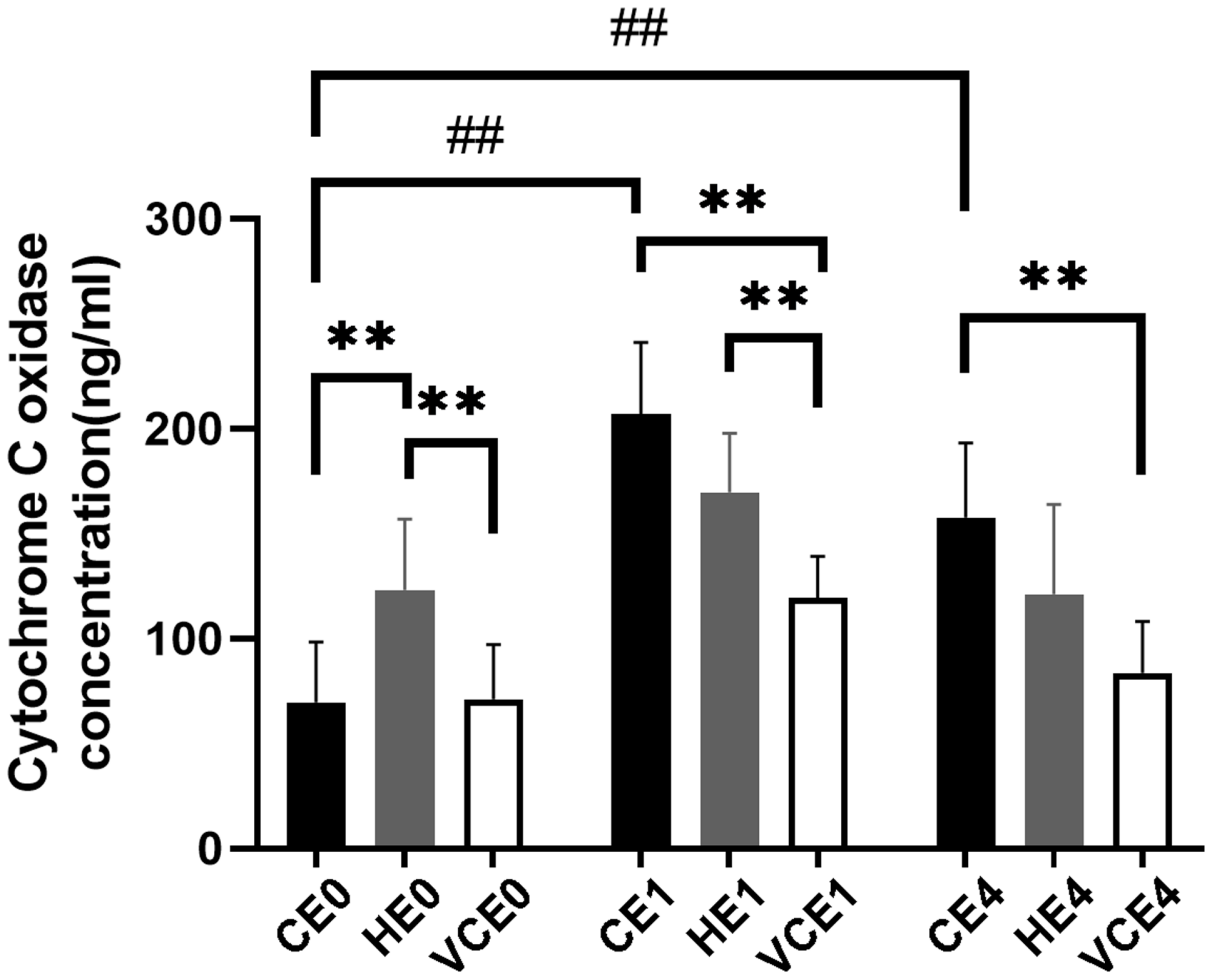
Changes in the level of the mitochondrial respiratory complex IV. C, blank control; H, inhalation of hydrogen gas; VC, vitamin C supplementation; E0, baseline; E1, immediately after exercise; and E4, 4h after exercise. *n*=8 per group. ** *p* < 0.01; ## *p* < 0.01.

Quantification of complex IV concentration in the gastrocnemius muscle found a main effect in both the exercise and intervention factors, as well as an interaction between the two factors (all *p* < 0.01). *Post hoc* tests identified a significant increase in complex IV concentration in both the CE1 and CE4 groups compared with the CE0 group (all *p* < 0.01). The intervention with vitamin C impeded this increase and resulted in a significant decrease in VCE1 and VCE4 compared with the CE1, HE1, and CE4 groups (all *p* < 0.01). This significant change was not seen after hydrogen inhalation, indicating that hydrogen intervention did not decrease complex IV concentration after exercise. Instead, the HE0 group showed a significantly elevated complex IV concentration relative to group CE0 and VCE0 (all *p* < 0.01; Figure 1). There was a decrease in complex IV concentration in each group at 4h after exercise compared to the group immediately after exercise, but none was statistically significant.

**Figure 2:**
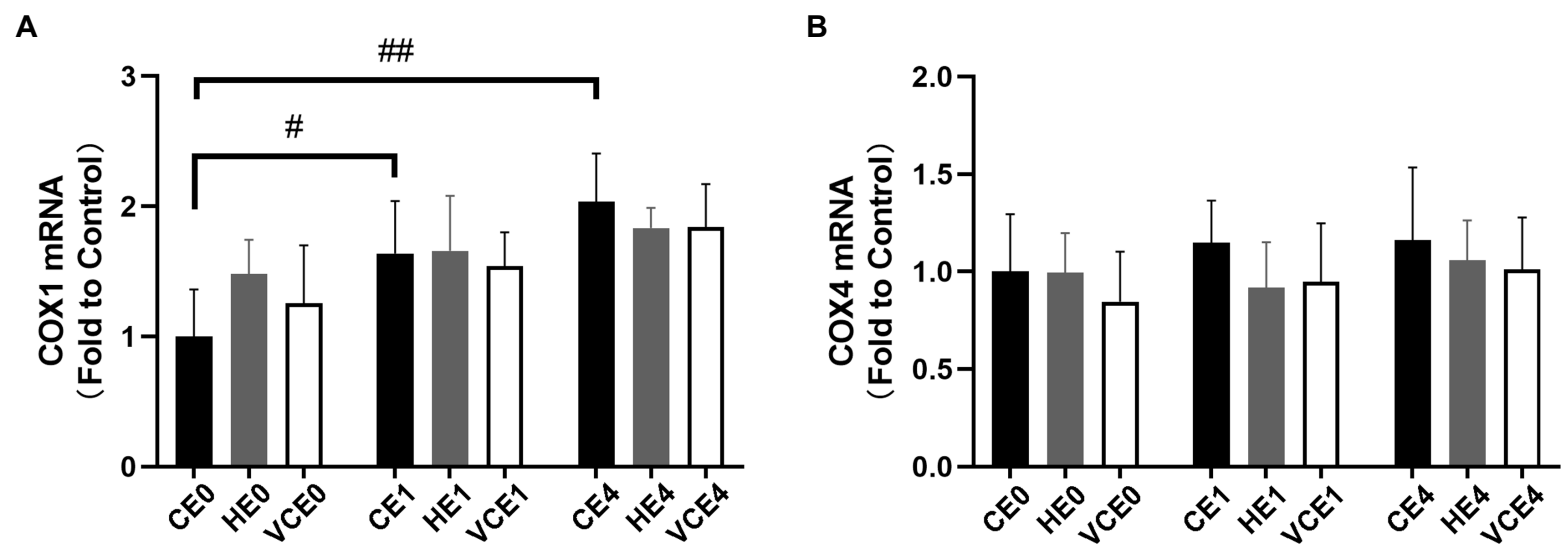
Average mRNA expression levels of COX1 **(A)** and COX4 **(B)** subunits. C, blank control; H, inhalation of hydrogen gas; VC, vitamin C supplementation; E0, baseline; E1, immediately after exercise; and E4, 4h after exercise. *n*=8 per group. #*p* < 0.05; ##*p* < 0.01.

Also, the COX1, a subunit of mitochondrial complex IV in skeletal muscle, showed a main effect of exercise on its mRNA transcription (*p* < 0.01), with that in the CE1 and CE4 was significantly increased compared with the CE0 (*p* < 0.05, *p* < 0.01, respectively), while no significant differences were found in COX4 subunit (Figure 2).

### Analysis of Mitochondrial Biogenesis and Signaling Pathways

**Figure 3:**
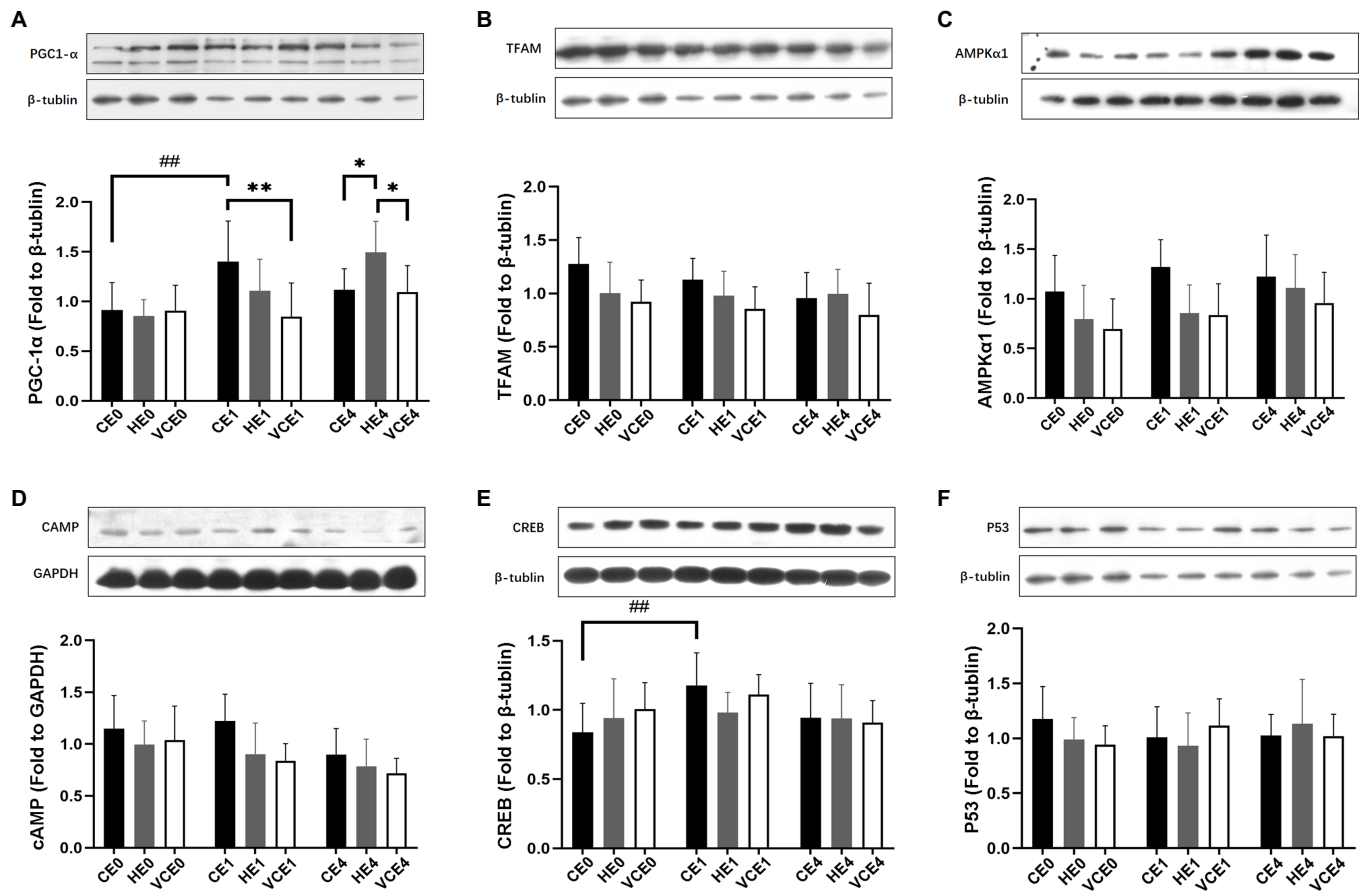
The protein expressions of PGC-1α **(A)**, TFAM **(B)**, AMPKα1 **(C)**, cAMP **(D)**, CREB **(E)**, and p53 **(F)** in gastrocnemius muscle, measured by western blot. C, blank control; H, inhalation of hydrogen gas; VC, vitamin C supplementation; E0, baseline; E1, immediately after exercise; and E4, 4h after exercise. *n*=9 per group. *p < 0.05, **p < 0.01; ##*p* < 0.01.

Analyses of PGC-1α, cAMP, TFAM and AMPK1α protein expression showed a main effect of intervention (PGC-1α and cAMP: *p* < 0.05, TFAM and AMPKα1: *p* < 0.01), PGC-1α, cAMP, AMPKα1 and CREB showed a main effect of exercise (PGC-1α and CREB: *p* < 0.01, AMPKα1 and cAMP: *p* < 0.05), and PGC-1α showed a significant interaction between the two factors (*p* < 0.01). Analysis of the protein expression found that the exercise induced a significant increase in CREB expression (*p* < 0.01; Figure 3E). The *post hoc* tests showed that the PGC-1α protein expression levels in the CE1 group was significantly increased relative to that in the CE0 group (*p* < 0.01), but vitamin C significantly attenuated the exercise-induced increase in PGC-1α expression (VCE1 vs. CE1: *p* < 0.01). Instead, the HE4 group showed a significant elevation relative to groups CE4 and VCE4 (all *p* < 0.05; Figure 3A). The protein expression of TFAM and cAMP showed a trend of reduction with vitamin C intervention compared to the control group, although these were not statistically significant (Figures 3B,D).

**Figure 4:**
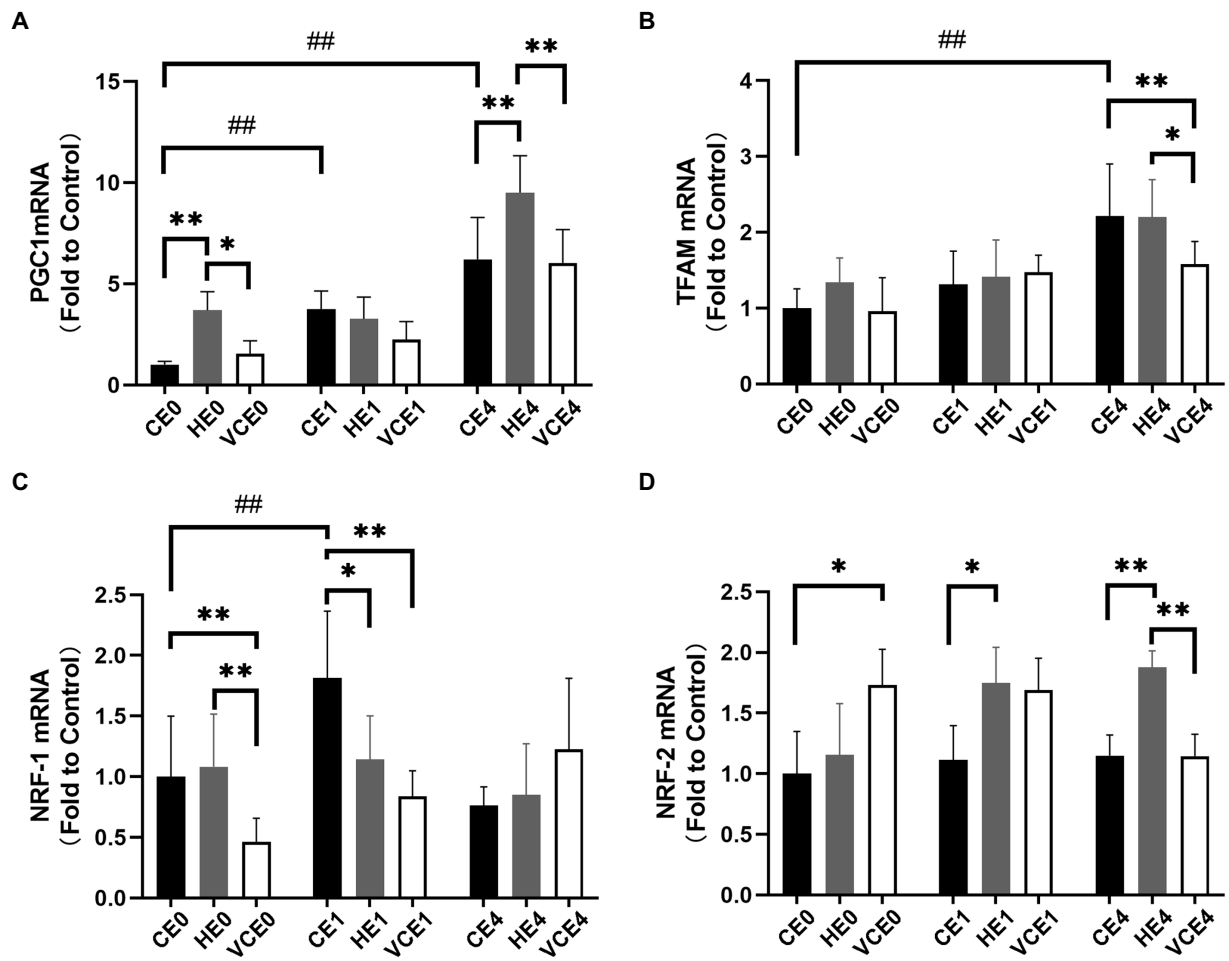
The mRNA expression levels of PGC-1α **(A)**, TFAM **(B)**, NRF-1 **(C)**, and NRF-2 **(D)** in gastrocnemius muscle. C, blank control; H, inhalation of hydrogen gas; VC, vitamin C supplementation; E0, baseline; E1, immediately after exercise; and E4, 4h after exercise. *n*=8 per group. * *p* < 0.05, **p < 0.01; ##*p* < 0.01.

The two-way ANOVA found that PGC-1α, NRF-1, and TFAM gene transcription had a significant main effect of intervention (NRF-1 and TFAM: *p* < 0.05, PGC-1α: *p* < 0.01) and exercise (all *p* < 0.01). PGC-1α and NRF-1 showed a significant interaction (*p* < 0.01). A single bout of exercise significantly increased the mRNA transcription of PGC-1α, TFAM, and NRF-1 genes (all *p* < 0.01). Hydrogen gas inhalation, with or without exercise, significantly increased gene transcription of PGC-1α compared with the control (CE0 vs. HE0, CE4 vs. HE4: all *p* < 0.01), as well as with the VC group (HE0 vs. VCE0: *p* < 0.05, HE4 vs. VCE4, *p* < 0.01). In addition, vitamin C supplementation significantly reduced mRNA transcription of TFAM (VCE4 vs. CE4 and HE4: *p* < 0.01, *p* < 0.05) and NRF-1 (VCE0 vs. CE0 and HE0, VCE1 vs. CE1: all *p* < 0.01), but this decrease was not seen in the hydrogen group (Figures 4A,B). The Kruskal–Wallis test showed that both antioxidant interventions significantly increased NRF-2 transcription in groups VCE0, and HE1 compared with CE conditions (all *p* < 0.05). The HE4 group still maintained a higher transcription levels relative to the CE4 and VCE4 groups 4h after exercise (all *p* < 0.01; Figure 4D).

### Changes in Endogenous Antioxidant Enzymes

**Figure 5:**
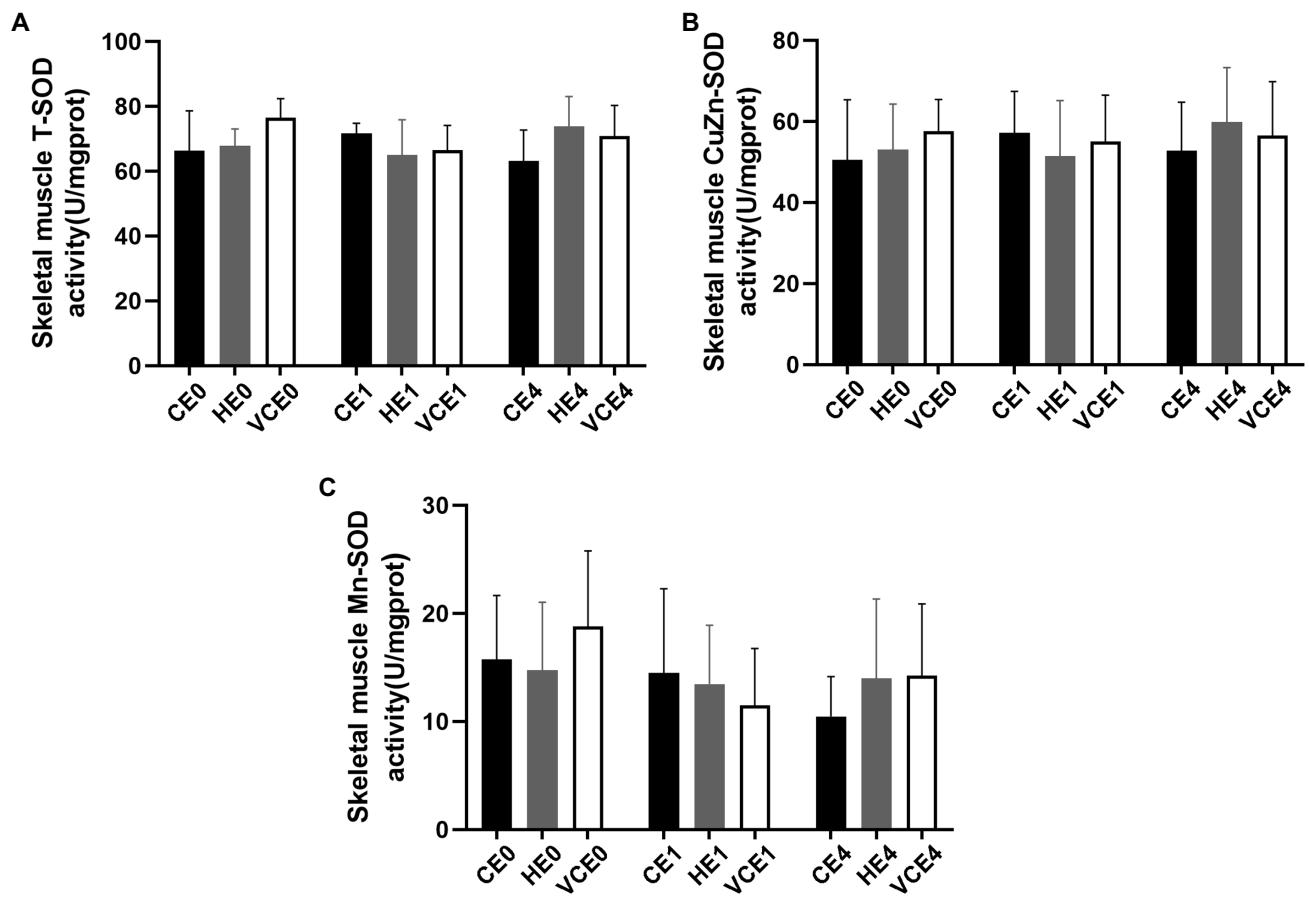
Total SOD (T-SOD) activity **(A)** and CuZn-SOD activity **(B)** in gastrocnemius muscle of each group, and Mn-SOD activity **(C)** was calculated according to their subtraction. C, blank control; H, inhalation of hydrogen gas; VC, vitamin C supplementation; E0, baseline; E1, immediately after exercise; and E4, 4h after exercise. *n*=8 per group.

**Figure 6:**
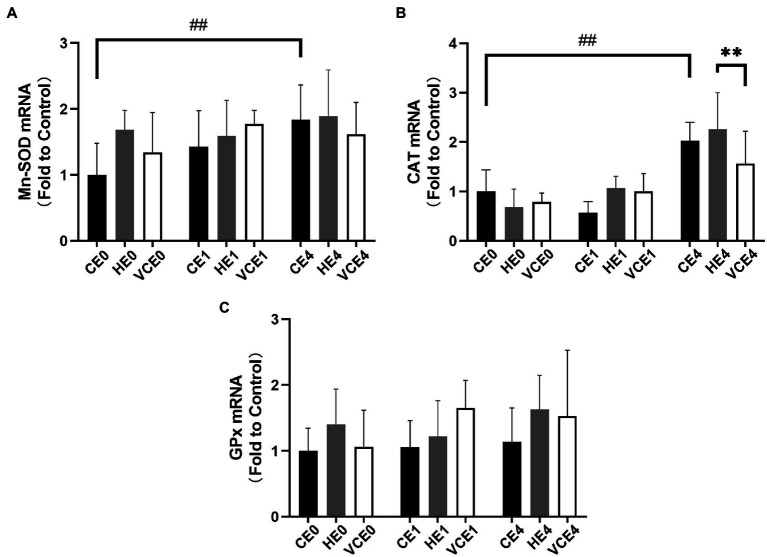
The mRNA expression levels of superoxide dismutase Mn-SOD **(A)**, CAT **(B)**, and glutathione peroxidase (GPx) **(C)** in gastrocnemius muscle detected by RT-qPCR. C, blank control; H, inhalation of hydrogen gas; VC, vitamin C supplementation; E0, baseline; E1, immediately after exercise; and E4, 4h after exercise. *n*=8 per group. ** *p* < 0.01; ##*p* < 0.01.

The Kruskal–Wallis test found no significant changes in the T-SOD, CuZn-SOD, and Mn-SOD (Figure 5). A two-way ANOVA of gene transcription of antioxidant enzymes showed a main effect of exercise for Mn-SOD and CAT (*p* < 0.05 and *p* < 0.01, respectively), and an interaction between the two factors for CAT (*p* < 0.01). There were no significant differences in the mRNA expression levels of Mn-SOD and GPx between the groups (Figures 6A,C). Four hours after exercise, the CAT transcription level was significantly increased in CE4 compared with CE0 (*p* < 0.01), and in HE4 compared with VCE4 (*p* < 0.01; Figure 6B).

### Biomarkers of Oxidative Stress

**Figure 7:**
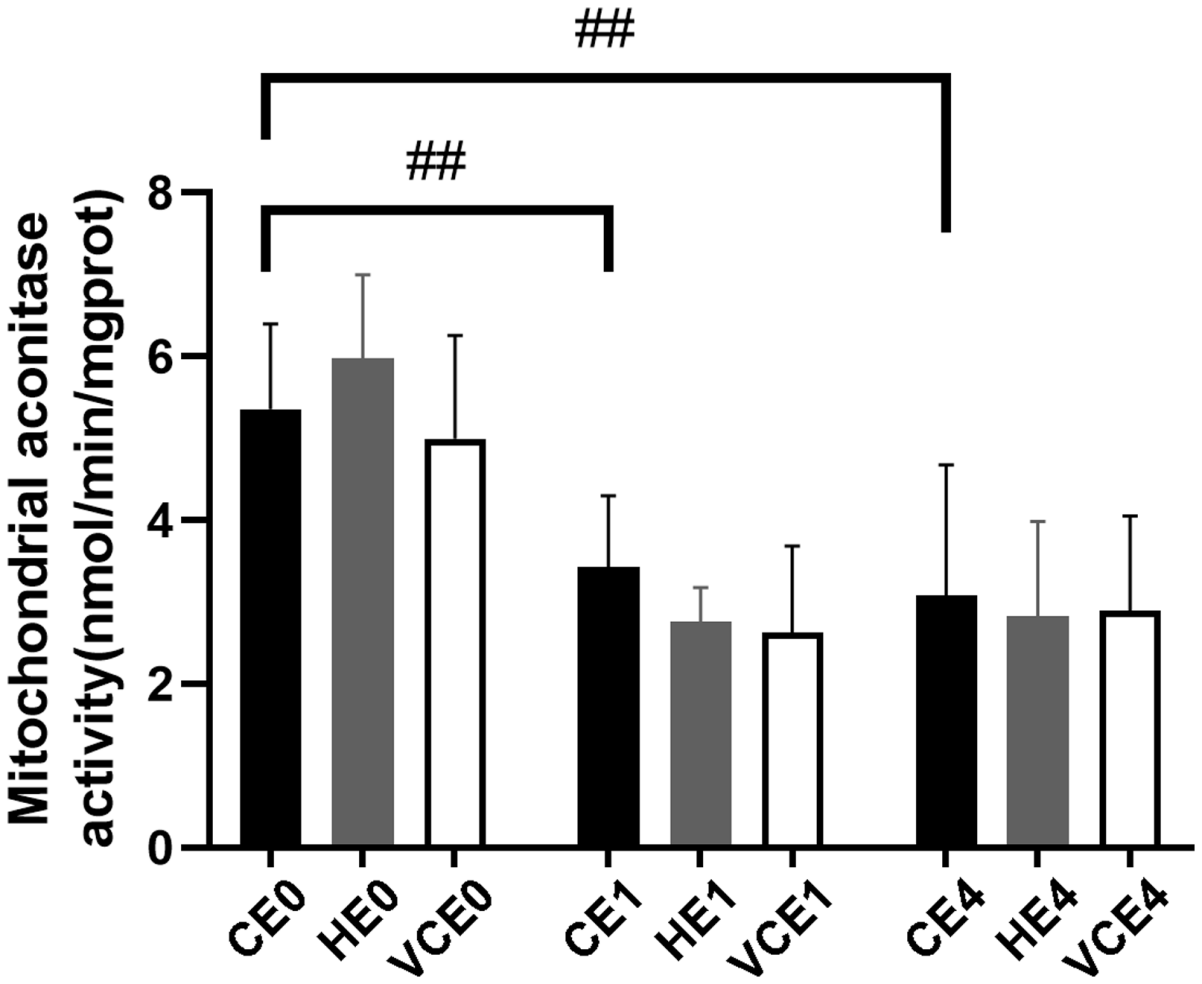
Changes of mitochondria aconitase activity in gastrocnemius muscle before and after exercise. C, blank control; H, inhalation of hydrogen gas; VC, vitamin C supplementation; E0, baseline; E1, immediately after exercise; and E4, 4h after exercise. *n*=8 per group ##*p* < 0.01.

**Figure 8:**
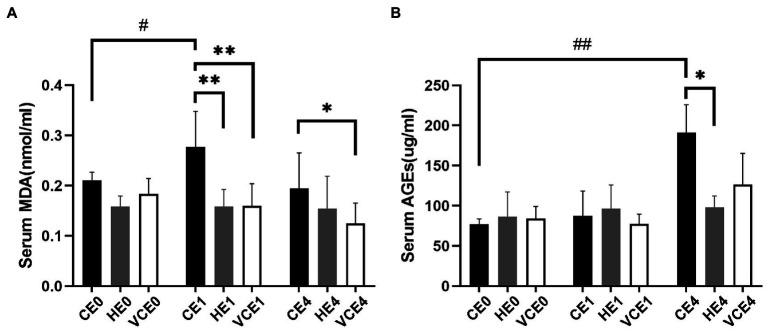
Lipid peroxidation products in serum, as markers of oxidative stress, were determined by detection of MDA **(A)**. The serum AGEs by ELISA indicates the extent of protein oxidation damage **(B)**. C, blank control; H, inhalation of hydrogen gas; VC, vitamin C supplementation; E0, baseline; E1, immediately after exercise; and E4, 4h after exercise. *n*=8 per group. *p < 0.05, **p < 0.01; #*p* < 0.05; ##*p* < 0.01.

The ACO activity in gastrocnemius muscle showed a main effect of exercise (*p* < 0.01). Exercise, with or without antioxidant supplementation, significantly decreased the activity of ACO in each group compared with non-exercise (CEO vs. CE1 and CE4: all *p* < 0.05; Figure 7). Serum MDA had a main effect of intervention (*p* < 0.05) and exercise (*p* < 0.01) as well showed an interaction between the two factors (*p* < 0.01). The serum MDA level significantly increased after exercise (CE0 vs. CE1: *p* < 0.05), and both hydrogen and vitamin C supplementations significantly reduced serum MDA level after exercise (CE1 and HE1 vs. VCE1: all *p* < 0.01, CE4 vs. VCE4: *p* < 0.05), (Figure 8A). The Kruskal–Wallis test for the serum level of AGEs found a significant increase at 4h after exercise (CE4 vs. CE0: *p* < 0.01), and the AGEs in the HE4 were significantly lower than those of the CE4 (*p* < 0.05; Figure 8B).

## Discussion

The effects of antioxidant supplementation before exercise have been controversial. A review by Peternelj and Coombes (2011) analysed 23 studies that showed that antioxidant supplements blunt the effects of exercise training of across different intensities and forms, although the oxidative stress levels were reduced (Peternelj and Coombes, 2011). It has been reported that the rats treated with Allopurinol-induced inhibition of oxidative stress demonstrated a reduced duration of exercise and expression of NF-κB, compared with control, during a treadmill test with the intensity progressively increased to exhaustion (Gomez-Cabrera et al., 2005). Vitamin C supplementation prevented mitochondrial biogenesis signalling after a 6-week moderate intensity training (5days/week, 75%VO_2_max, 25→85min/day; Gomez-Cabrera et al., 2008). It has also been reported that, when rats’ spinotrapezius muscle was electrically stimulated to produce twitches (1Hz twitch contractions for 180s), blood flow was significantly reduced in the VC supplement group compared with control (Copp et al., 2009). These are all partially attributed to the exercise-induced ROS being removed by antioxidants (Powers et al., 2020).

There has been evidence that hydrogen gas has a selective antioxidant capacity that will only scavenge the strongly oxidative ^•^OH and will not scavenge ^•^O_2_^−^ (Ohsawa et al., 2007), therefore the H_2_O_2_ resulting from ^•^O_2_^−^ disproportionation will also not be affected. It has been speculated that hydrogen supplementation can retain the signal transduction capacity of ROS while reducing oxidative damage (LeBaron et al., 2019). Because there is a redundancy in organismal mitochondrial biogenesis activation signals and the effects of antioxidants may be offset by long-term training (Ristow and Zarse, 2010; Merry and Ristow, 2016a), in this study we used an acute exercise model to compare the antioxidant effects between vitamin C supplementation and H_2_ inhalation.

In our study, it was found that pre-exercise inhalation of hydrogen reduced the levels of oxidative stress markers. We compared mitochondrial ACO activity and serum concentrations of oxidative stress markers between groups. ACO is a key enzyme in the tricarboxylic acid cycle, located in the inner mitochondrial membrane in close proximity to the complexes on the membrane. An increased ROS will decrease the activity of aconitase, and this activity is commonly used to reflect the changes in ROS (Liu et al., 2013; Tsui et al., 2013). Furthermore, MDA and AGEs were commonly used markers to reflect lipid and protein peroxidation (Marrocco et al., 2017). In this study, the MDA and AGEs in the serum increased after exercise (*p* < 0.05) which might be related to the exercise-induced ROS. However, the serum MDA and AGEs in the H_2_ inhalation group were significantly lower than those in the exercise-only group (*p* < 0.05), that would indicate that hydrogen gas had played a role in reducing oxidative stress (Figure 8).

As a strong antioxidant, vitamin C acts *via* a reversible dehydrogenation reaction and rapidly reacts with ^•^O_2_^−^, HOO^−^, and ^•^OH to produce dehydroascorbic acids, scavenge singlet oxygen, and reduce sulfur radicals. As a hydrogen donor, vitamin C can also exert an indirect antioxidant effect by oxidizing vitamin E and sulfhydryl groups to their reduced form (Janisch et al., 2005). In this study we found that when rats were supplemented with vitamin C, the amount of PGC-1α protein, NRF-1 and TFAM mRNA expression in the gastrocnemius muscle was significantly reduced compared with the control and hydrogen groups (Figures 3A, 4A,B). PGC-1α is an upstream activator of mitochondrial biogenesis in muscle that affects ROS production, but at the same time its expression is also regulated by the ROS. Therefore, there potentially exists a regulatory interaction between PGC-1α and ROS during exercise. After vitamin C having scavenged a large amount of ROS, the effect of PGC-1α as well as the expression of downstream signaling molecules are blocked, ultimately leading to blunted post-exercise mitochondrial biogenesis. This is underscored by the lower complex IV concentration post exercise in the VCE1 and VCE4 groups than that in the CE1 and CE4 groups (Figure 1).

Notably, hydrogen inhalation did not blunt the mitochondrial biogenesis process following the exercise as did the vitamin C supplementation, nor did it affect the key signaling molecules activated by ROS. After hydrogen inhalation, these signaling molecules were significantly higher than that in the VC intervention group (Figures 4B,C). Even more interestingly, hydrogen inhalation also directly activated PGC-1α. The PGC-1α mRNA in the HE0 and HE4 groups, and PGC-1α protein in the HE4 group were significantly increased compared with that in the CE and VCE groups (Figures 3A, 4A). PGC-1α plays a central role in mitochondrial biogenesis and antioxidant enhancement through several signaling kinases, including AMPK, p53, and cAMP (Scarpulla, 2011). In addition, another study found that the expression of HO-1 and Sirtuin-1 (Sirt1) mRNA and Sirt1 protein in primary hepatocytes increased, subjected to hydrogen saline treatment, and especially, Sirt1 was obviously upregulated (Li et al., 2018). Sirt1 is a NAD^+^-dependent deacetylase, and an over-expression of Sirt1 promotes the deacetylation of PGC-1α, which is an activated state of PGC-1α in the activation process of mitochondrial biogenesis (Rodgers et al., 2005). Sirt1 cooperates with AMPK pathway to construct Sirt1/AMPK-PGC-1α-TFAM axis in regulation and promotion of the mitochondrial function (Thirupathi and de Souza, 2017). Therefore, we hypothesize that hydrogen inhalation could activate PGC-1α at the gene and protein levels that leads to a higher complex IV concentration, as shown in the HE0 group compared to CE0 and VCE0 (Figure 1), that may result in an increased level of mitochondrial biogenesis to some extent (Timón-Gómez et al., 2018).

We speculate that the inhalation of hydrogen gas did not affect ^•^O_2_^−^ and H_2_O_2_ (Ohsawa et al., 2007; Sakai et al., 2019), nor to having reduced the beneficial ROS-mediated mitochondrial biogenesis, as compared with the vitamin C supplementation. Furthermore, the hydrogen inhalation did not cause a significantly higher expression of the selected proteins (Figure 3), as compared with that of the vitamin C supplementation. The specific signal proteins require further study.

In addition, we also found that both hydrogen and vitamin C supplementation significantly increased the mRNA transcription of NRF-2 in skeletal muscle (Figure 4D). NRF-2 and its endogenous inhibitor, Keap1, function as a ubiquitous, evolutionarily conserved intracellular defence mechanism to counteract oxidative stress (Merry and Ristow, 2016b; Bellezza et al., 2018). Hydrogen has been shown to activate the NRF-2 signaling pathway (Fang et al., 2020). The intensity of the 1h endurance exercise was not enough to cause significant change in the activity of NRF-2-regulated antioxidant enzyme, SOD, neither in the GPx gene expression. However, after 4h recovery, the CAT mRNA was significantly higher in both the CE4 and HE4 groups, and in the HE4 group than that in the VCE4 group (Figure 6B), this may be owing to the higher NRF-2 expression maintained at 4h after the hydrogen+exercise intervention.

Although various types of ROS were not directly measured in this study, the signal molecules downstream of ROS and the key proteins upstream of PGC-1α were measured. It was found that the expression of AMPKα1 and cAMP proteins showed a trend of decrease in both the HE and VCE groups (not significantly), and there was no significant difference in p53 protein between the interventions (Figure 3). The changes in the upstream signal of mitochondrial biogenesis directly controlled by ROS in response to different ROS and antioxidants need to be explored in future studies.

We could not measure the concentration of hydrogen in the gastrocnemius and blood during exercise after hydrogen inhalation due to unavailability of specific equipment, so it is difficult to confirm the real-time dynamic changes of hydrogen in the tissues in relation to the antioxidant responses. A recent study found that hydrogen inhaled by pigs (one breath only) diffused into tissues (advection diffusion), with the H_2_ concentration in the arterial blood decreased quickly within 3min after inhalation, but that in the venous blood decreased in a slower rate and remained more or less higher than the baseline level even 60min after inhalation, indicating that the tissues had retained and or metabolized some of the H_2_ (Sano et al., 2020). We speculate that the antioxidant benefits during exercise could be due to either the residual influence of the tissue reactions during the 60min H_2_ inhalation, or the effect of the H_2_ retained in tissues after inhalation, or both. To our knowledge, this study was the first to investigate the acute effect of 1-h hydrogen inhalation 30min before a bout of endurance exercise. Further investigations are required to explore the pharmacokinetics of H_2_ intervention and verify these hypotheses.

Given that hydrogen gas inhalation prior to exercise did not affect mitochondrial biogenesis as much as vitamin C supplementation, but induced an increased expression of PGC-1α as found in this study, and has no known side effects, hydrogen gas certainly holds promise as an effective antioxidant.

## Limitations

Some limitations in this study should be considered in interpretation of the findings and construction of future studies. In this study, only the gastrocnemius muscle was analyzed. This muscle consists of a high proportion of the fast twitch, glycolytic muscle fibres, therefore its metabolic changes and oxidative stress might be different to those of the muscles consisting of predominantly slow twitch, oxidative fibres. It was the first study to investigate the effect of hydrogen inhalation at 4%, 30min prior to a treadmill exercise. The actual H_2_ concentration in the blood and tissues was not monitored during and after inhalation. The potential effect of hydrogen inhalation at other time points or dosages before or during exercise might result in different responses. Finally, the actual levels of various ROS in the exercising muscle were not assessed, but only the selected markers in the relevant regulatory pathways. Future studies are required to examine these issues.

## Conclusion

Breathing air with 4% hydrogen and ingesting vitamin C at a dosage of 500mg/kg, prior to 1-h endurance treadmill exercise, both resulted in a significantly decreased aconitase activity in the gastrocnemius muscle, associated with an increased levels of serum MDA and AGEs. These would indicate that hydrogen inhalation could be an effective antioxidant in the management of oxidative stress in exercise. The advantages of hydrogen inhalation could be that it did not blunt the mitochondrial biogenesis process as did the vitamin C supplementation in response to the exercise. Inhalation of hydrogen 30min before exercise appeared to increase the mitochondrial complex IV concentration, activate PGC-1α, TFAM, and NRF-2 gene transcription, and up-regulate PGC-1α protein expression level.

## Data Availability Statement

The original contributions presented in the study are included in the article/Supplementary Material; further inquiries can be directed to the corresponding authors.

## Ethics Statement

The animal study was reviewed and approved by Animal Care and Use Committee of Tianjin University of Sport, Tianjin, China (approval number TJUS2019023).

## Author Contributions

LC and WL designed the experiments. LC conducted most of the experiments, analyzed the data, and wrote the manuscript. ZY helped to collect the samples and conduct the experiments. WL and ZS supervised the study, designed the experiments, and revised the manuscript. YZ contributed to translation of the manuscript. All authors contributed to the article and approved the submitted version.

## Conflict of Interest

The authors declare that the research was conducted in the absence of any commercial or financial relationships that could be construed as a potential conflict of interest.

## Publisher’s Note

All claims expressed in this article are solely those of the authors and do not necessarily represent those of their affiliated organizations, or those of the publisher, the editors and the reviewers. Any product that may be evaluated in this article, or claim that may be made by its manufacturer, is not guaranteed or endorsed by the publisher.
